# Dual-Readout Self-Resetting CMOS Image Sensor for Resolving Sub-Percent Optical Contrast in Biomedical Imaging

**DOI:** 10.3390/s26041396

**Published:** 2026-02-23

**Authors:** Kiyotaka Sasagawa, Subaru Iwaki, Kenji Morimoto, Ryoma Okada, Hironari Takehara, Makito Haruta, Hiroyuki Tashiro, Jun Ohta

**Affiliations:** 1 Division of Materials Science, Graduate School of Science and Technology, Nara Institute of Science and Technology, 8916-5 Takayama, Ikoma 630-0192, Nara, Japan; 2 Medilux Research Center, Nara Institute of Science and Technology, 8916-5 Takayama, Ikoma 630-0192, Nara, Japan; 3 Institute for Research Initiatives, Nara Institute of Science and Technology, 8916-5 Takayama, Ikoma 630-0192, Nara, Japan; ohta@ms.naist.jp; 4 Department of Opto-Electronic System Engineering, Chitose Institute of Science and Technology, 758-65 Bibi, Chitose 066-8655, Hokkaido, Japan; 5 Department of Health Sciences, Faculty of Medical Sciences, Kyushu University, 3-1-1 Maidashi, Higashi-ku 812-8582, Fukuoka, Japan

**Keywords:** CMOS image sensor, self-resetting pixel, high dynamic range, dual-readout architecture, artifact reduction, signal-to-noise ratio, biomedical imaging, microcirculation

## Abstract

We report a dual-readout self-resetting CMOS image sensor that achieves a signal-to-noise ratio (SNR) exceeding 70 dB and resolves sub-percent optical contrast variations by effectivly suppressing reset artifacts. The proposed sensor employs a Dual-Readout architecture with two independent scanners operating with a temporal offset; while one readout system is in the self-reset “dead time”, the other remains active, thereby physically ensuring continuous data acquisition. To minimize pixel area while achieving high reconstruction accuracy, a minimum frame-to-frame difference algorithm is utilized for signal restoration without requiring in-pixel counters. A prototype chip fabricated in a 0.35-μm process demonstrated SNR characteristics near the shot-noise limit, with a peak SNR exceeding 70 dB. Vascular phantom experiments using a carbon black suspension successfully visualized ±0.25% contrast fluctuations—dynamic signals previously undetectable by conventional sensors. This device provides a powerful platform for high-precision bio-imaging applications, including brain surface blood flow monitoring, where both wide dynamic range and high SNR are essential.

## 1. Introduction

Non-invasive and long-term imaging of physiological dynamics in vivo, such as cerebral blood flow and neural activity, is essential for the advancement of neuroscience and clinical diagnosis. Unlike fluorescence imaging, which uses strong exogenous indicators, detecting weak signals such as hemodynamic responses under natural behavior or changes in voltage-sensitive dyes is extremely difficult [1,2,3,4,5,6,7,8]. These target signals often exhibit only minute intensity modulations of 0.1% to 1% against strong background light. To reliably resolve such minute fluctuations without being buried in noise, an image sensor with an extremely high signal-to-noise ratio (SNR), typically exceeding 70 dB, is required.

**Figure 1:**
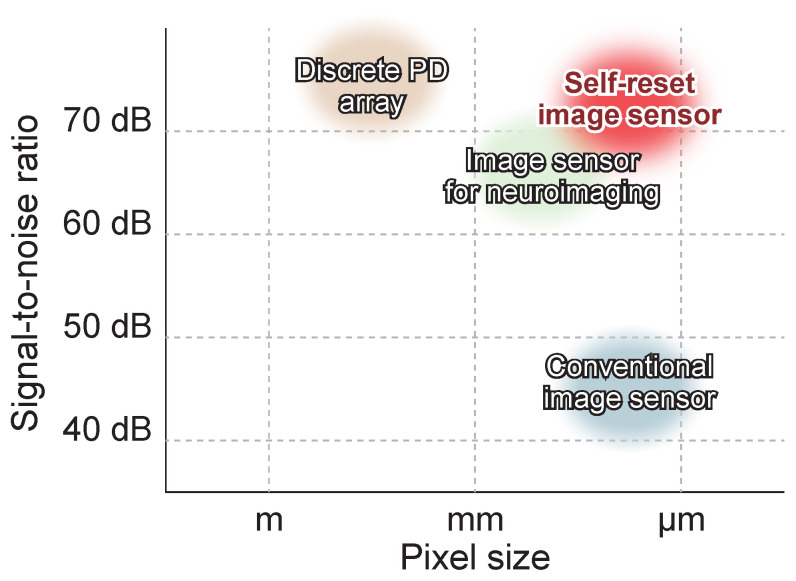
Conceptual comparison of the propsed image sensor with other image sensors.

However, meeting this high SNR requirement with a standard CMOS active pixel sensor (APS) faces fundamental physical constraints, as shown in Figure 1. Optical signals associated with brain activity are typically small, with a fractional intensity change (ΔI/I) typically ranging from 0.1% to 1.0% [1,2]. To resolve such minute modulations with high fidelity and quantitative accuracy, a system-level signal-to-noise ratio (SNR) exceeding 70 dB is indispensable. This high SNR is physically limited by shot noise, necessitating a high photon flux of approximately $10^{7}$ photons per pixel. The maximum SNR, limited by photon shot noise, is determined by the pixel’s full well capacity (FWC). Reducing pixel size to meet demands for high spatial resolution or compact devices proportionally decreases FWC, significantly limiting the achievable SNR. Therefore, SNRs exceeding 70 dB have typically been achieved using large pixels or photodiode arrays rather than standard fine pixels [9,10,11]. However, this introduces a significant trade-off between resolution and signal quality.

In recent years, methods using special pixel structures such as LOFIC (lateral overflow integration capacitor) pixels have been developed to overcome FWC limitations in fine pixels [12,13,14,15], but these require specialized CMOS processes. In contrast, asynchronous self-resetting (or folding integration) pixel architectures can be realized with standard CMOS processes. By automatically resetting the integration node upon saturation and counting the number of resets, the effective FWC is increased, enabling high dynamic range (HDR) and high SNR even with fine pixels [16,17,18,19,20].

Previously, we proposed ultra-compact imaging devices for biological use by designing CMOS image sensors [21]. While small microscopes mountable on a mouse head have been developed [22,23,24], designing a dedicated image sensor allows for further miniaturization and weight reduction. Accordingly, we developed compact and high-SNR imaging using self-reset sensors for intrinsic imaging and voltage-sensitive dyes [25,26,27].

However, a critical defect compromising signal integrity remained: the “reset artifact”. The finite dead time required for asynchronous reset operation leads to a temporary loss of incident photon information. In conventional readout methods, this periodic information loss appears as a sharp drop in SNR, or an “SNR dip”, at the folding boundaries of the input–output characteristics. For high-precision bio-imaging, this artifact is catastrophic, as the target minute signals (fluctuations of approximately 0.1%) risk being completely obscured by noise fluctuations at these boundaries.

In this paper, we propose a self-resetting CMOS image sensor with significantly reduced artifacts, designed specifically for the detection of minute biological signals. To eradicate reset artifacts, we propose a novel Dual-Readout Architecture and a simple reconstruction algorithm. By sampling the asynchronous output from a 3T-based self-resetting pixel via two independent readout paths with an intentional time offset, complementary signal acquisition is ensured. That is, even if one path encounters a reset dead time, the other captures a valid continuous signal. This method, combined with a complementary reconstruction algorithm using the dual-readout results, achieves a seamless response with high SNR. This enables the detection of ultra-minute optical contrasts of less than 1%, which were previously buried in the noise floor, even in dynamic environments.

The main contributions of this study are summarized as follows:

- A pixel circuit combining a standard 3T structure with an in-pixel Schmitt trigger self-reset circuit for stable asynchronous operation.
- A novel readout architecture that achieves non-destructive interleaved sampling with a time offset using two row-selection scanners, significantly reducing reset dead time artifacts.
- A robust off-chip reconstruction framework incorporating FPN correction and a minimum frame-to-frame difference algorithm to restore highly linear images.
- Experimental verification using a prototype chip. Using a vascular phantom mimicking biological tissue, we demonstrated extremely wide dynamic range operation involving up to 15 resets. Furthermore, we confirmed high SNR characteristics capable of clearly visualizing minute luminance changes (density fluctuations) of ±0.25% even in dynamic flow, ensuring measurement reliability.

The remainder of this paper is organized as follows. Section 2 describes the mechanism of reset artifact generation and the concept of the proposed architecture to resolve it. Section 3 reports the circuit implementation details of the prototype chip and the fundamental characterization of the single pixel. Section 4 presents the details of the reconstruction algorithm and a quantitative evaluation of the artifact reduction effect by system application. Section 5 presents a microfluidic demonstration using a biomimetic phantom, demonstrating high reliability in dynamic imaging and superior detection capability for minute density fluctuations. Finally, Section 6 concludes this paper.

## 2. Proposed Dual-Readout Architecture

In this section, we define the mechanism of reset artifact generation, which is a critical issue in self-resetting pixels. We then describe the concept of the “Dual-Scanner Interleaved Readout” architecture proposed in this study to fundamentally resolve this issue.

### 2.1. Definition of Reset Artifacts

Although the self-resetting (folding integration) operation is a powerful method for preventing pixel saturation and extending the effective FWC, it inevitably introduces a period during which signal continuity is lost due to its operating principle.

**Figure 2:**
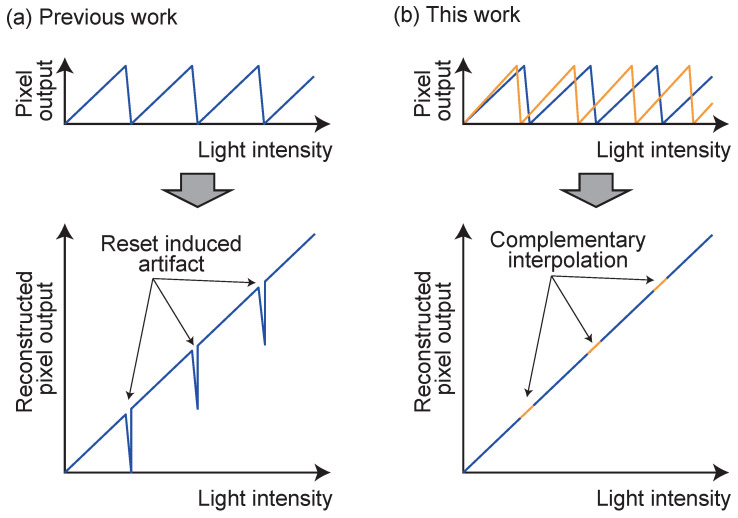
(**a**) Output characteristics of a self-resetting pixel. Artifacts occur during reconstruction. (**b**) Output characteristics of the dual-readout self-resetting pixel. Artifacts are avoided by using two outputs complementarily.

Figure 2a (top) shows the ideal output response of a self-resetting pixel to a constant optical input. When the comparator within the pixel detects the saturation level (threshold), the pixel enters a self-reset operation, draining the accumulated charge and returning the value to its initial state. A finite dead time (dead time: $t_{dead}$), determined by the circuit response speed, occurs from the start of this reset operation until its completion and the resumption of stable integration.

During this period, the pixel becomes temporarily “blind” to incident light, and the integration of signal charge is interrupted. In self-resetting pixels where a single row-selection scanner performs sampling at a fixed period, if the sampling timing accidentally coincides with this dead time, a meaningless value during integration or an unstable potential during the reset transient is read out. This is the “reset artifact”. This loss of information occurs periodically under specific light intensity conditions where the readout timing coincides with the reset timing, as the reset period changes according to the incident light intensity. If the number of self-resets can be counted, the light intensity value can be reconstructed from the self-reset pixel output. However, as a result of this artifact, a dip occurs in the reconstructed output.

### 2.2. Concept of Dual-Scanner Readout

To eliminate the aforementioned reset artifacts, it is necessary to guarantee that sampling is not performed during the reset dead time. However, since self-resets occur asynchronously, it is difficult to predict their timing in advance to control the readout. Therefore, in this study, we propose a complementary dual-readout method that utilizes the non-destructive readout characteristic of CMOS image sensor pixels without a floating diffusion.

Figure 2b shows the conceptual diagram of the proposed method. Since non-destructive readout is performed twice, the pixel output values differ under the same light intensity condition, creating a difference in the points where self-reset occurs. Consequently, even under conditions that would cause a dip in one readout, a continuous signal can be obtained in the other. By combining both complementarily, a reconstructed signal without dips can be obtained.

**Figure 3:**
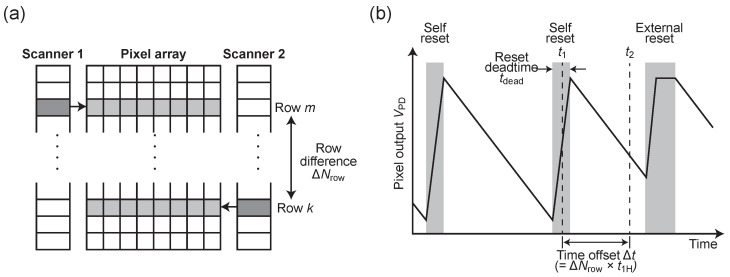
(**a**) Conceptual diagram of two scanners scanning with a row difference Δy. (**b**) Principle of complementary sampling: if the time difference Δt caused by the row difference is longer than the dead time $t_{dead}$, one sampling is always valid.

Figure 3a shows the implementation form in the image sensor. In this architecture, two independent readout pointers (Scanner 1 and Scanner 2) are provided for the pixel array. These are driven by the same clock and scan while maintaining a constant row address difference (Δy). In the rolling shutter operation, this row address difference Δy translates directly into a difference in access time (time offset Δt) for a specific pixel. That is, the same pixel is read out non-destructively twice: at time $t_{1}$ (by Scanner 1) and at time $t_{2}=t_{1}+\Delta t$ (by Scanner 2).

There is an important design guideline for setting this time offset Δt. First, as a fundamental requirement, Δt must be set sufficiently longer than the pixel’s reset dead time $t_{dead}$ ($\Delta t>t_{dead}$). As shown in Figure 3b, this guarantees that even if one readout timing (e.g., $t_{1}$) coincides with the dead time resulting in signal loss, the other timing ($t_{2}$) will invariably fall within a period avoiding the dead time.

Furthermore, in this study, Δt is optimized so that the two readout results have a “complementary relationship” within the target light intensity range. That is, the offset is set so that when one readout is near the reset boundary (low SNR region), the other is located near the center of the integration period (high SNR region). By adaptively selecting and synthesizing the valid data from these two complementary sets, significant mitigation of reset artifacts is achieved. While the temporal offset Δt could theoretically introduce motion artifacts in the case of rapid sample movement, such effects are typically negligible at high frame rates and could be further compensated for by temporal interpolation or image registration techniques in the signal processing stage.

In this study, the temporal offset Δt is fixed rather than adaptive to simplify the hardware implementation. Although the integration period varies with illumination intensity, a fixed Δt is sufficient to ensure that the light intensities triggering reset artifacts (due to overlap with the dead time) differ between the two readout channels. Therefore, at any given intensity, at least one channel consistently provides valid data, which is then selectively utilized by the reconstruction algorithm to maintain high SNR across a wide range.

## 3. Circuit Implementation and Pixel Characterization

This section describes the implementation details of the CMOS image sensor chip fabricated to realize the architecture proposed in Section 2, as well as its fundamental characterization. First, the outline of the prototype chip is presented, followed by the specific implementation of the pixel circuit. We also present the results of experiments to optimize the pixel operating conditions (bias voltage). Furthermore, we demonstrate that the dual readout is performed as intended by the prototype image sensor.

### 3.1. Prototype Chip Overview

**Figure 4:**
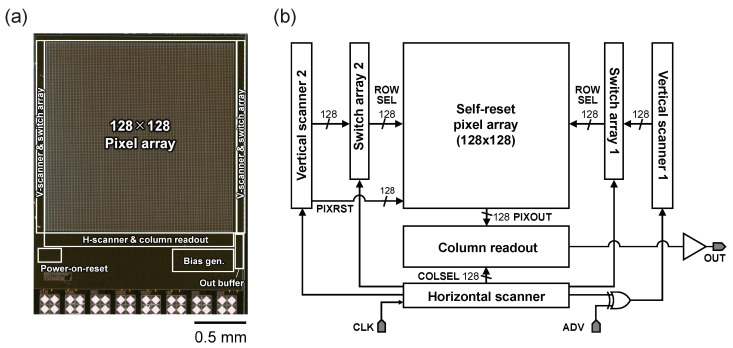
(**a**) Micrograph of the test chip fabricated in 0.35 μm CMOS process. (**b**) Circuit block diagram.

To demonstrate the effectiveness of the proposed method, an image sensor chip was fabricated using a standard 0.35 μm CMOS process. Figure 4 shows a micrograph of the prototype chip. The chip integrates a self-resetting pixel array and peripheral circuits including the dual scanners described later. The two switch array blocks act as a time-division multiplexer. During one horizontal period, the switch arrays select either vertical scanner 1 or 2 based on the timing control, effectively routing the corresponding ROWSEL signal to the pixel array. This configuration allows a single shared column readout circuit to process the non-destructive outputs from two different rows sequentially within the same frame. Regarding the COLSEL signals shown in Figure 4b, they are primarily used to control the column-multiplexers in the column readout block to pick specific column data for the output buffer. Although the diagram shows COLSEL crossing the pixel array, this is for routing simplification; the COLSEL signal does not participate in the pixel-level integration or reset processes. Table 1 summarizes the main specifications of the prototype chip. The pixel pitch is 15 μm. The analog signals from the pixel array are digitized using an external 14-bit analog-to-digital converter (ADC). The input voltage range of the ADC is set to 1.92 V, resulting in a quantization step (1 LSB) of approximately 120 μV. Given the pixel’s analog output swing of approximately 1.2 V, the signal is resolved into approximately 10,240 digital levels within the 14-bit range.

### 3.2. Circuit Implementation

#### 3.2.1. Pixel Circuit

**Figure 5:**
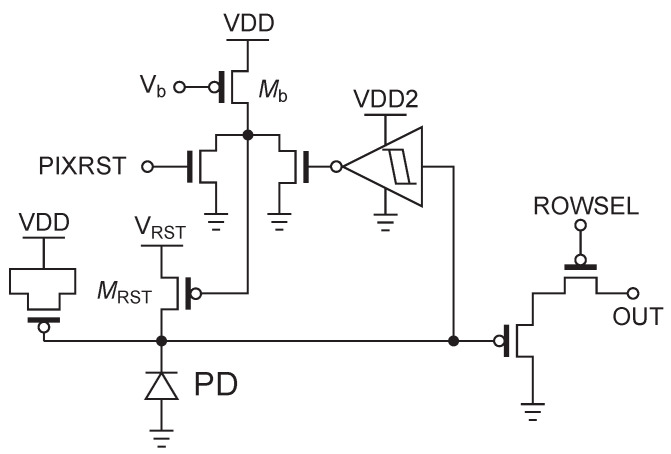
Circuit configuration of the 3T-based self-resetting pixel with an asynchronous reset loop. $M_{RST}$: Reset transistor, $M_{b}$: Reset period adjustment transistor. PIXRST: External reset, ROWSEL: Row selection.

Figure 5 shows the circuit diagram of the pixel used in this study. This pixel is based on a standard 3-transistor (3T) active pixel sensor (APS) structure combined with an in-pixel self-reset circuit [28,29]. While many self-resetting pixels incorporate a counter within the pixel to count the number of self-resets [16,20], this pixel does not include a counter. This allows for a larger proportion of the photodiode (PD) area within the pixel.

The 3T-APS base structure consists of a PD for photoelectric conversion and signal charge integration, a source follower transistor (SF) for buffering the PD node voltage ($V_{PD}$), and a select transistor (SEL) for row selection. In this configuration based on 3T-APS, there is no floating diffusion (FD), and $V_{PD}$ is directly input to the gate of the SF. Here, the source follower and selection switch are composed of pMOS transistors to effectively utilize the low voltage range.

The self-reset circuit section consists of a comparator that monitors the PD node voltage and a reset transistor ($M_{RST}$) controlled by its output. The comparator is composed of a low-power Schmitt trigger inverter and an inverter for buffering. To suppress the through-current that flows when the Schmitt trigger inverter switches, $V_{DD2}$ is set to 1.9 V, which is lower than $V_{DD}$ [26].

Additionally, this pixel aims to measure with a high signal-to-noise ratio (SNR) in a region where photon shot noise is dominant, using stronger light that makes normal image sensor pixels saturate. Although the effective capacity is extended by self-reset, an increase in the number of self-resets increases the probability of artifact occurrence. Therefore, a pMOS capacitor is mounted in parallel with the PD to physically extend the pixel capacity and design for more stable operation.

Regarding pixel operation, when $V_{PD}$ drops due to incident light and falls below the comparator threshold, a reset operation is automatically performed. At this time, the gate voltage of the transistor ($M_{b}$) in the comparator can be adjusted externally to control the reset period. This allows adjustment of the period during which $M_{RST}$ is on.

The reset voltage $V_{RST}$ of 2.4 V is supplied from an external source. During self-reset, the voltage needs to return to the reset voltage in a short time. By connecting an EMI filter to the external $V_{RST}$ supply line to suppress the drop in reset voltage, the SNR degradation during self-reset is improved [27].

#### 3.2.2. Dual Scanner Peripheral Circuit

To realize the time-offset readout described in Section 2, two independently controllable row selection scanners (Scanner 1 and Scanner 2) are arranged on both sides of the pixel array. Both scanners operate synchronously with the same master clock that controls the entire chip, but are designed to scan while always maintaining a constant row address difference Δy by controlling their start timing (or initial address). This simple control makes it possible to generate a precise sampling time difference Δt for each pixel while minimizing the circuit scale.

### 3.3. Pixel Characterization

#### 3.3.1. Optimization of Operating Conditions

The length of the reset dead time ($t_{dead}$) and the stability of operation in the self-reset action strongly depend on the bias condition ($V_{b}$) of the current-limiting pMOS transistor ($M_{b}$) included in the in-pixel self-reset circuit. Optimizing the bias condition of $M_{b}$ is essential to minimize the influence of reset artifacts and realize stable operation.

**Figure 6:**
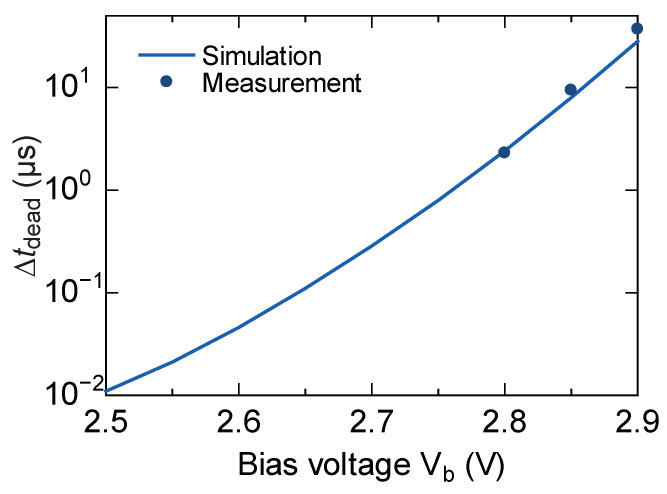
Dependence of reset dead time $t_{dead}$ on pixel bias voltage $V_{b}$. The good agreement between measured and simulated values indicates that exponential control is possible.

First, the dependence of the reset dead time $t_{dead}$ on the bias voltage $V_{b}$ was measured, and the results are shown in Figure 6. The solid line represents the simulation results, and the dots represent the measured results. As $V_{b}$ increases, the drive current of $M_{b}$ decreases and the reset operation becomes slower, so $t_{dead}$ tends to increase exponentially. Measurements were limited to $V_{b}\geq 2.8\;V$ due to the bandwidth limitations of the output buffer circuit, which prevented the detection of shorter pulse widths. However, within the measurable range, the measured results agree very well with the simulation, confirming the validity of the simulation model and that the dead time can be controlled widely and precisely by $V_{b}$.

**Figure 7:**
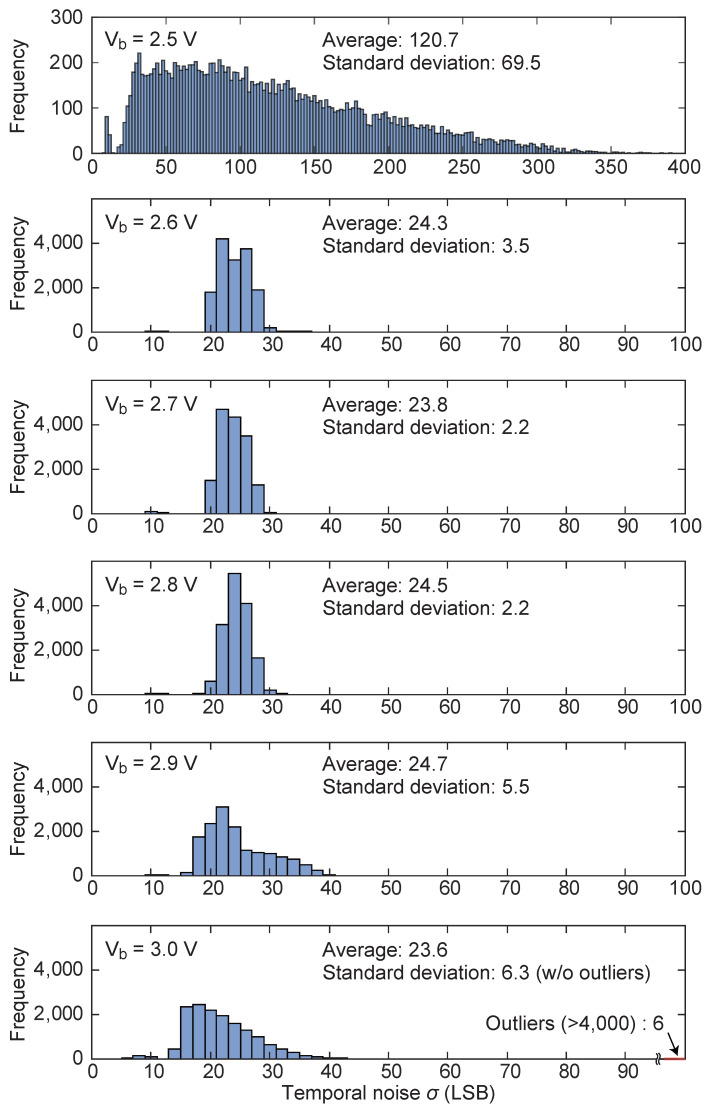
Histograms representing the frequency distribution of temporal noise magnitude (σ) across the pixel array at different bias voltages $V_{b}$. The x-axis indicates the temporal noise in LSB (1 LSB ≈ 117.2 μV), and the y-axis indicates the number of pixels. At $V_{b}=2.7\;V$, both the average noise and its variation across the array are minimized, indicating the most stable and uniform operation.

Next, to evaluate the stability of the self-reset operation, we analyzed the temporal noise across the entire pixel array. Temporal noise is defined as the standard deviation (σ) of the output values for a single pixel, calculated over 1000 consecutive frames under constant illumination. To visualize the uniformity and magnitude of this noise, we calculated σ for each of the 16,384 pixels (128 × 128 array) and plotted their frequency distribution as a histogram in Figure 7. In these plots, the x-axis represents the magnitude of the temporal noise in LSB, and the y-axis represents the number of pixels exhibiting that noise level. Figure 7 shows the noise distribution at different $V_{b}$. Under low-bias conditions such as $V_{b}=2.5\;V$, the average value of temporal noise is extremely high at 120.7 LSB. This increased temporal noise is attributed to the incomplete settling of the integration node during the self-reset process. At this higher bias voltage, the increased speed of the comparator results in an extremely narrow self-reset pulse. This pulse width is insufficient for the integration node potential to settle completely at the target reset level ($V_{RST}$). Consequently, the stochastic variation in the initial potential of each integration cycle introduces additional temporal noise. This phenomenon stems from the insufficient settling time caused by the high-speed feedback response, rather than a simple feedback delay.

On the other hand, under a high-bias condition of $V_{b}=3.0\;V$, the standard deviation of the noise distribution across the array increases significantly to 84.8 LSB. While the majority of pixels follow a distribution similar to the $V_{b}=2.9\;V$ case, the presence of six extreme outliers with noise exceeding 4000 LSB drastically inflates the overall statistical spread. As noted in Figure 7, the standard deviation becomes 6.3 LSB when these six outliers are excluded, which is comparable to the other bias conditions. These outliers are attributed to highly unstable reset cycles at this bias point, where the integration node fails to settle correctly.

In the range of $V_{b}$ = 2.6∼2.8 V, both the average noise and its variation are kept low, indicating that stable self-reset operation is obtained. Based on these results, we selected $V_{b}=2.7\;V$ as the optimal operating point for this study, as it provides high operational stability and keeps $t_{dead}$ sufficiently short at approximately 200 ns, and used it for subsequent measurements.

#### 3.3.2. Pixel Output Characteristics

**Figure 8:**
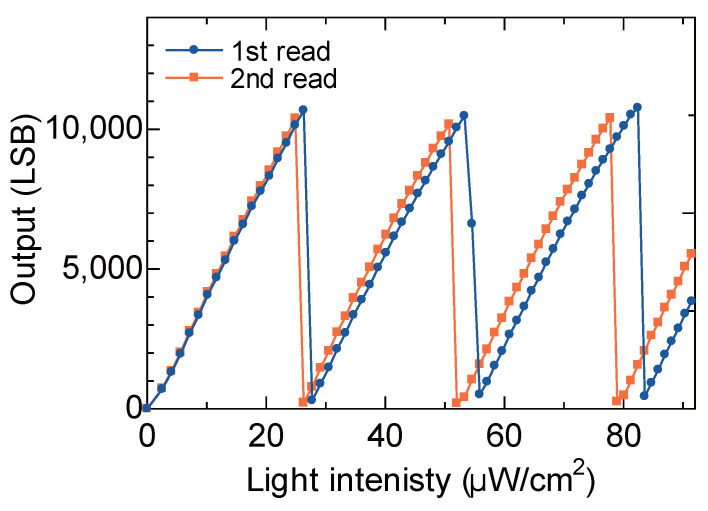
Pixel output characteristics versus irradiation light intensity.

Under the optimal operating conditions selected in the previous section, the fundamental photoelectric conversion characteristics of the prototype pixel were evaluated. The results are shown in Figure 8. Each point is the average pixel output obtained from 1000 frames acquired continuously at a frame rate of 30 frames/s. It can be seen that the self-reset operation is occurring and that self-reset is triggered at different light intensities due to the two readouts.

In this measurement result, at the point of irradiation light intensity 55 μW/cm^2^ in the first readout, the measurement point overlaps with the self-reset boundary, taking an intermediate value. At this point, it is inferred that the number of self-resets differs for each frame, resulting in instability. In such a case, an SNR dip will occur.

When performing imaging, such points inevitably occur to some extent within the imaging region. At such points, reset artifacts occur even if optimization such as bias adjustment of the single pixel is performed. In other words, this suggests that a fundamental solution to avoid this is indispensable for detecting weak signals.

## 4. Reconstruction Algorithm and System Evaluation

In this section, we detail the off-chip reconstruction algorithm for restoring highly linear images without artifacts from the two analog raw data streams ($V_{1},V_{2}$) acquired by the dual-scanner readout. We also present the results of basic operation verification and SNR characteristics measurement when applying this system, quantitatively proving that the proposed method effectively eliminates reset artifacts and breaks through the physical limits of SNR in fine pixels.

### 4.1. Minimum Frame-to-Frame Difference Reconstruction Algorithm

The output of a self-resetting image sensor is a signal folded by self-reset. Several groups have proposed reconstruction algorithms for such signals [30,31,32]. In this study, taking advantage of the characteristic that signal intensity changes are very small in the target bio-measurements (such as blood flow and neural activity), we propose a unique and simple reconstruction algorithm based on the minimal frame-to-frame difference principle.

**Figure 9:**
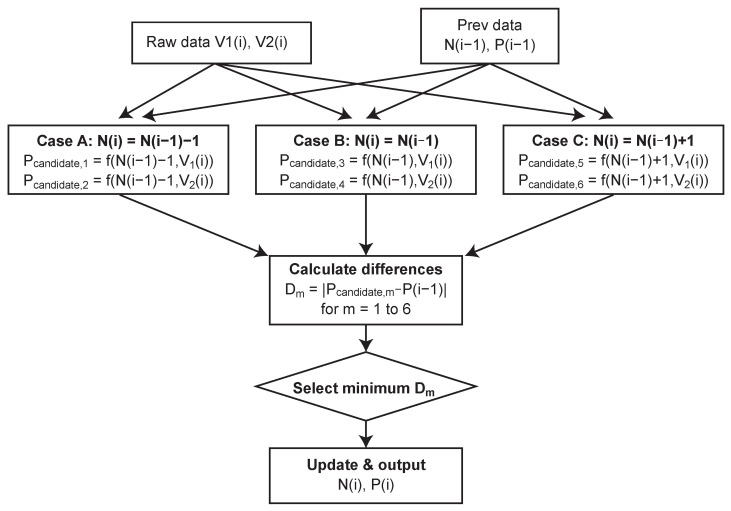
Processing flow of the proposed reconstruction algorithm based on the minimum frame-to-frame difference principle. The value closest to the previous frame is selected from six candidates.

Figure 9 shows the processing flow of the proposed algorithm. The processing is performed in the following steps.

#### 4.1.1. Preprocessing

First, to remove fixed pattern noise (FPN) caused by variations in the Schmitt trigger threshold and source follower characteristics for each pixel, standard correction using a pre-acquired dark image is applied to all raw data. In addition, linearization processing to correct minute non-linearities in the self-reset pixel using response characteristics to light intensity is also applied at this stage. This process uses a lookup table generated from pre-acquired non-linear response characteristics. Also, since there is a sensitivity difference due to the difference in exposure time between the first and second readouts, gain correction is performed as a temporal normalization based on the integration time ratio. Since both readouts share the same signal path and the timings are precisely controlled by the system clock, accurate correction is achieved.

#### 4.1.2. Reset Count Estimation and Sample Selection

Next, the number of resets is estimated, and samples free from artifacts are selected. Here, given a reset count *n* and a sample voltage *v*, the function f(n,v) calculating the linearized signal value *P* is defined as follows:

(1) $$f\left(n,v\right)=n\cdot P_{step}+\left(P_{sat}-v\right)$$

where $P_{step}$ is the signal step amount corresponding to one reset, and $P_{sat}$ is the saturation level value. In the target biomedical applications, the sensor is integrated into a system with a controlled light source. By gradually increasing the illumination intensity from complete darkness (N=0), the system ensures that the change in the reset count between consecutive frames does not exceed one during the ramp-up phase. This controlled initialization allows the sensor to continuously and reliably track the cumulative number of self-resets, thereby establishing a valid starting point for the tracking algorithm without the need for pre-acquired reference images.

The algorithm assumes that the reset count N[k] in the current frame *k* is either “maintained”, “+1”, or “−1” relative to the confirmed value $N_{final}\left[k-1\right]$ of the previous frame (changes of ±2 or more are excluded due to the slow signal change). Based on each possibility and the combination of the two sample voltages ($V_{1},V_{2}$), a total of six candidate values $P_{candidate}$ are generated:

- Case A (*N* decreased): $f(N_{final}\left[k-1\right]-1,V_{1})$, $f(N_{final}\left[k-1\right]-1,V_{2})$
- Case B (*N* maintained): $f(N_{final}\left[k-1\right],V_{1})$, $f(N_{final}\left[k-1\right],V_{2})$
- Case C (*N* increased): $f(N_{final}\left[k-1\right]+1,V_{1})$, $f(N_{final}\left[k-1\right]+1,V_{2})$

Then, among these candidate values, the one with the minimum absolute difference from the final confirmed value of the previous frame $P_{final}\left[k-1\right]$ is adopted as the most plausible true value $P_{final}\left[k\right]$ for the current frame. Through this selection process, even if one of the samples (e.g., $V_{1}$) is an abnormal value during the reset dead time, the candidate value based on it will have a large difference from the previous frame and will be automatically rejected, ensuring that the appropriate sample and correct reset count combination are always selected.

### 4.2. Demonstration of Basic Operation

To verify the dynamic reconstruction capability of the proposed algorithm, time-series data were acquired when the incident light intensity to the sensor was changed in a triangular wave shape over time.

A green LED (Thorlabs ML530L3, center wavelength 530 nm) was used as the light source, and the incident light intensity was swept by increasing and decreasing its drive current in 2 mA steps within the range of 0 mA to 500 mA. The sensor frame rate was set to 30 fps, and images were acquired at each step. The selection row offset of the left and right scanners was set to 5 rows.

**Figure 10:**
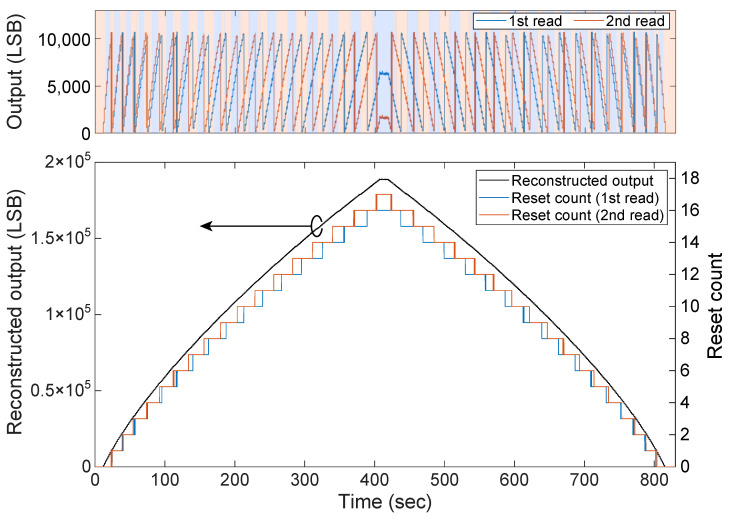
Results of signal reconstruction experiment for time-varying light input. (**Top**) Raw data from dual scanners. Background color indicates the selection result by the algorithm (Blue: 1st readout adopted, Red: 2nd readout adopted). (**Bottom**) Reconstructed output signal (black line) and estimated reset count (colored line). The reset count is correctly estimated even without a counter, and a continuous waveform is obtained.

The experimental results are shown in Figure 10. The top panel of the figure shows the waveforms of the two raw data streams ($V_{1}$ and $V_{2}$) obtained from the dual scanners. As the incident light intensity changes, the output value is repeatedly folded (folding) by the self-reset operation. Also, it can be confirmed that a phase difference is generated between the two waveforms due to the time offset as designed. Under this condition, the number of self-resets is about 15, and it can be seen that the phase is exactly inverted.

The background color of the graph indicates which data the reconstruction algorithm adopted. The light blue region indicates that the first readout data ($V_{1}$) was selected, and the light red region indicates that the second readout data ($V_{2}$) was selected as sound data free from artifacts.

The bottom panel of the figure shows the result of applying the proposed algorithm to these raw data. As shown on the right axis, the estimated reset count (*N*) changes stepwise according to the light intensity, correctly counting up to about 17 times, and then counting down.

The reconstructed output signal (solid black line) shown on the left axis is restored as a continuous waveform corresponding to the increase and decrease in the input light intensity, without causing steps or omissions even at the boundaries where the reset count switches (folding points).

This result demonstrates that by using the proposed method, it is possible to correctly estimate the reset count by tracking the slowly changing light intensity without having a reset counter in the pixel, and to reconstruct the signal by automatically avoiding invalid samples affected by the reset dead time.

However, if the incident light intensity fluctuates significantly and the self-reset count changes by more than ±1, correct results cannot be obtained. To cope with this, a more complex restoration algorithm is required. However, since this study specializes in detecting minute changes, no breakdown of the reconstructed image is observed in the demonstration shown in Section 5. Also, the measurable luminance change rate can be expanded by designing a larger pixel capacity.

### 4.3. Quantitative Evaluation of Extended FWC and Artifact Reduction

**Figure 11:**
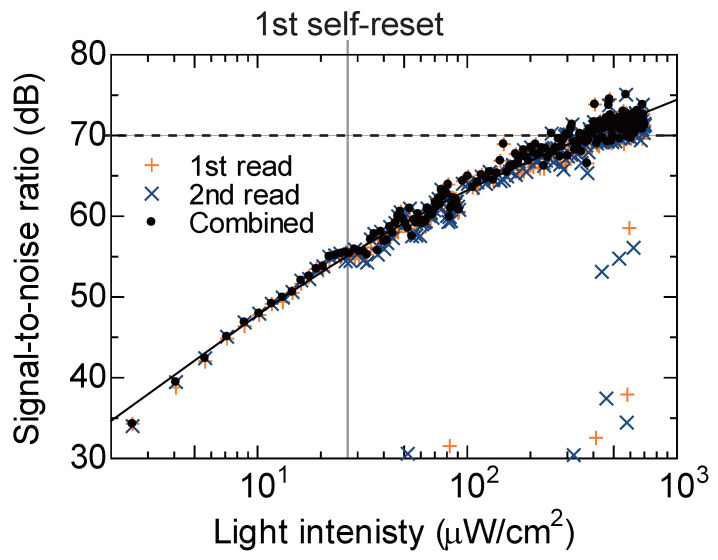
Signal-to-noise ratio of pixel output versus irradiation light intensity.

To quantitatively evaluate the final performance of the proposed system, particularly the effect of removing reset artifacts, the SNR characteristics were measured over a wide range of light intensities. The experimental results are shown in Figure 11.

In the figure, the orange “+ (1st read)” and blue “× (2nd read)” are the SNRs calculated using only one of the dual scanner outputs. This corresponds to the conventional single readout method. Confirming these plots, although the SNR generally improves with increasing light intensity, it can be seen that SNR dip points, where the SNR drops sharply locally, occur at light intensities corresponding to specific analog output values (near reset boundaries). This indicates the loss of information due to the coincidence of the readout timing with the reset dead time, as described in Section 2.

Here, the locations of the SNR dips differ between the first readout (+) and the second readout (×). Strictly speaking, if the measurement range is widened, there will be points where both overlap, but in cases where the brightness range to be measured is limited as in this study, the overlap can be avoided by appropriate setting. The designed time offset Δt functions appropriately, creating a state where when one is in the dead time, and the other is always in a stable integration period.

The black “• (Combined)” is the final SNR characteristic after applying the proposed reconstruction algorithm. For the reasons mentioned above, the algorithm adaptively selects sound samples from the two readout results, so the dips seen in the individual readouts have disappeared.

Focusing on the shape of this reconstructed SNR curve, it can be seen that the slope is 20 dB/decade in the low light intensity region and changes to 10 dB/decade in the high light intensity region. This indicates that the dominant factor of noise transitions from readout circuit noise (constant with respect to signal) in the low illuminance range to photon shot noise (proportional to the square root of the signal) in the high illuminance range.

Usually, self-reset operation involves noise penalties such as the addition of reset noise, but as already mentioned, in this study, the reset voltage ($V_{RST}$) from outside the chip is stabilized by using an EMI filter [27]. Although this configuration does not suppress the kT/C noise originating from the reset transistor (MRST), it is essential for stabilizing the reset potential against transient voltage drops during high-speed self-reset operations. By maintaining power supply integrity through an external source and an EMI filter, the sensor ensures a consistent starting potential for each integration cycle, thereby preserving linearity and temporal noise performance.

As a result, the SNR characteristics show a smooth response over the entire dynamic range, and a high SNR exceeding 70 dB, which is the target value for bio-measurement [1,2], was achieved in the high illuminance region. This result quantitatively proves that the proposed system fundamentally solves the reset artifact, which was an inherent problem of the self-reset method, and realizes high SNR in fine pixels.

Furthermore, based on the fitting results of this response curve and the point where the first self-reset occurs, the charge capacity corresponding to one self-reset is estimated to be about 0.78 Me^−^.

Based on the measured SNR characteristics, the dynamic range (DR) of the proposed sensor was estimated to be approximately 92 dB by extrapolating the noise floor and considering the maximum intensity achieved through 17 self-reset operations. Although this DR is sufficient for the target biomedical application, it remains lower than some of the state-of-the-art sensors listed in Table 2. This is primarily because our design is based on a 3-Tr active pixel sensor architecture, where kT/C noise (reset noise) is the dominant factor in the readout noise floor. This architectural choice was made to prioritize pixel simplicity and process versatility, which are crucial for the targeted implantable and wearable diagnostic devices.

## 5. Demonstration of Minute Change Imaging Using Vascular Phantom

### 5.1. Experimental Setup and Sample Preparation

**Figure 12:**
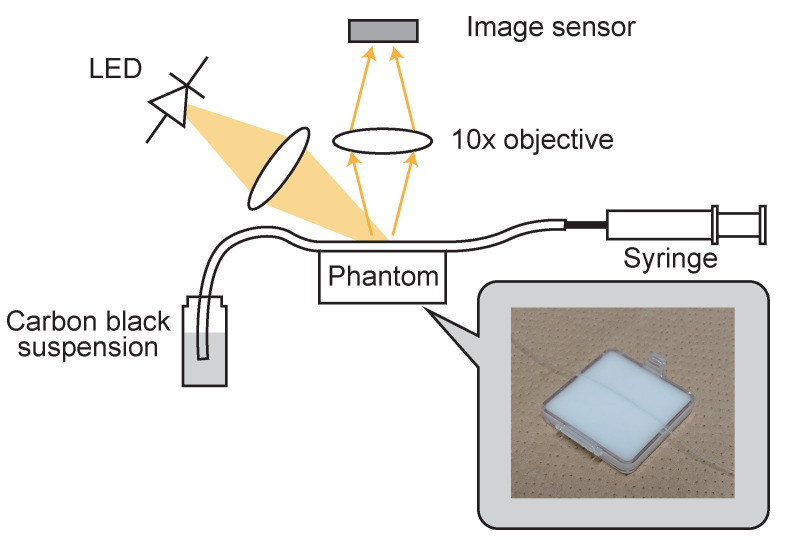
Imaging experimental setup using a microtube-based vascular phantom.

To evaluate the performance of the proposed sensor in a dynamic environment, a reflection-type microscopic observation experimental system shown in Figure 12 was constructed. A high-power LED (Thorlabs M590L4) with a peak wavelength of 590 nm, suitable for the absorption characteristics of hemoglobin, was used as the light source. A 10× objective lens was used, but the back focus distance (BFD) was intentionally shortened to about 20 mm. This non-standard optical configuration is an optimization to maximize the amount of light received on the sensor surface to realize a high SNR state where frequent self-resets occur, and to meet the demand for miniaturization required for future portable diagnostic devices and in vivo imaging of small animals under free behavior.

To simulate microvessels and surrounding tissues, a biomimetic phantom containing scatterers was prepared. To reproduce the optical scattering characteristics of tissues, rutile-type TiO_2_ powder was mixed into PDMS (polydimethylsiloxane). Specifically, 0.2 g of TiO_2_ powder was mixed with about 5 mL of ethanol, treated in an ultrasonic cleaner for 3 min to disperse uniformly, and then added to the silicone elastomer to cure so that the final TiO_2_ concentration was about 0.1 wt%. A PFA (perfluoroalkoxy alkane) microtube with an inner diameter of 0.3 mm was embedded in this scattering medium as a vascular phantom. A mixture of India ink (carbon black suspension) and saturated saline was used as the fluid sample. Due to the interaction with the saline solution, the carbon particles disperse unevenly, generating minute density fluctuations and aggregates that mimic the aggregation of red blood cells (rouleaux formation) and mottled patterns observed in actual microvessels.

### 5.2. Basic Performance of Wide Dynamic Range Imaging

**Figure 13:**
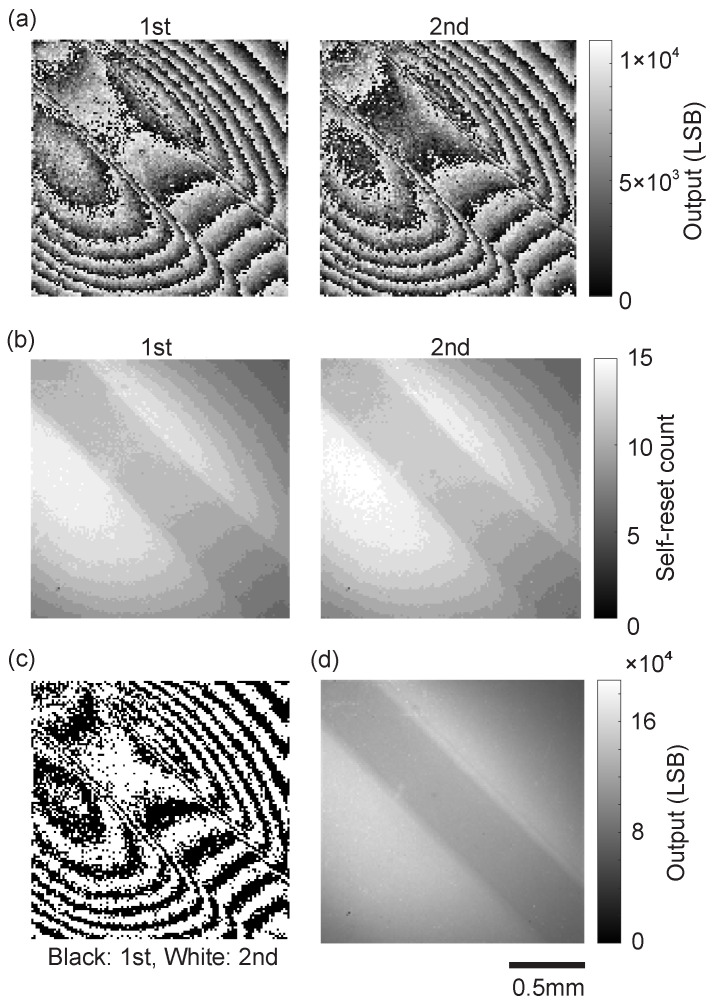
Flow channel phantom observation results by dual-readout self-resetting image sensor. (**a**) Raw images. (**b**) Extracted self-reset counts. (**c**) Readout selection map indicating the chosen source (Black: 1st, White: 2nd) for each pixel. (**d**) Reconstructed image.

The imaging results under strong illumination conditions are shown in Figure 13. Similar to the evaluation in the previous section, the LED current was gradually increased from 15 mA to 300 mA in 1 mA steps to accurately count the number of self-resets.

Figure 13a shows the raw images of the two readout results. The microtube is visible from the top left to the bottom right of the screen. Also, it can be seen that striped patterns appear because the luminance information is folded by self-reset. In addition, the second readout shows a slightly brighter imaging result. Figure 13b shows the result of extracting the distribution of self-reset counts for each. At the maximum light intensity irradiation, the self-reset count reached 15 in the brightest region. Figure 13c shows the distribution determining which image information (1st or 2nd) was used to create the composite image. Black dots indicate that the first readout result was used, and white dots indicate that the second readout result was used. Figure 13d is the image obtained by synthesis. If there are pixels with reset count judgment errors or dead states, they will have values significantly different from surrounding pixels. However, it can be seen that there are no such pixels in this image, and a smooth and continuous image is obtained.

This demonstration confirmed that the proposed sensor can maintain high linearity and robust operation over a wide dynamic range without image breakdown even under extremely strong light where conventional pixels would be completely saturated, and that the proposed reconstruction method can be applied to the entire imaging area without problems.

### 5.3. Reliability in Dynamic Imaging and Capability to Detect Minute Signals

**Figure 14:**
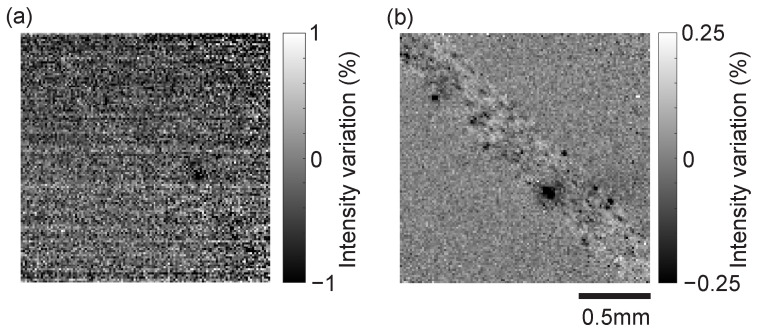
Difference images from the reference image. (**a**) Result under low illumination conditions where self-reset does not occur. (**b**) Result under high-illumination conditions with a maximum self-reset count of 15.

We verified the effectiveness of the proposed dual-readout architecture for dynamic flow. To observe weak changes, a difference image between a reference image and the observed image was acquired. Here, the reference image was created by frame-averaging 500 consecutive acquired images. Figure 14 shows the results under (a) irradiation light intensity where self-reset does not occur, and (b) the condition of Figure 13 (maximum self-reset occurs 15 times). Video recordings of these dynamic results are available in Supplementary Videos S1 and S2.

Under low-illumination conditions (Figure 14a), the readout noise becomes the dominant noise source relative to the low signal level, making its impact more visually apparent. Accordingly, the display range for the fractional change is set to ±1%. In this result, although the flow of large lumps of India ink is visible, finer details are obscured by the readout noise floor and can hardly be confirmed.

Under high-illumination conditions (Figure 14b), photon shot noise becomes dominant. From the results of Figure 11, the signal-to-noise ratio is in the region of about 70 dB, and the range of change rate is displayed as ±0.25%. In this image, it is possible to confirm the distribution of fine “hazy” mottled patterns caused by the concentration gradient. Also, in the moving image, it can be seen how this flows. Although this is the result of synthesizing two images, no breakdown at the self-reset boundary is clearly seen in this result, suggesting that synthesis has been performed with sufficiently high precision.

This result suggests the possibility that the proposed system can resolve slight fluctuations in red blood cell distribution and turbulence in blood flow in minute biological blood vessels, demonstrating a significant advantage in high-precision biomedical imaging.

## 6. Conclusions

In this study, we proposed an artifact-suppressed self-resetting CMOS image sensor to capture minute biological signals such as red blood cell aggregation in microvessels. By combining the proposed “dual-readout architecture” and the “minimum frame-to-frame difference reconstruction algorithm”, we succeeded in dramatically suppressing the artifact during self-resetting, which was unavoidable in conventional self-resetting sensors.

In the dynamic fluid imaging demonstration using a vascular phantom, the following results were obtained. First, we demonstrated that image reconstruction maintaining high linearity over a wide dynamic range is possible without image breakdown by the proposed algorithm even in an extreme environment where a maximum of 15 self-resets occur under strong illumination conditions. Second, we confirmed that the proposed method effectively suppresses reset artifacts and has high measurement reliability for dynamic objects involving extremely minute luminance changes of ±0.25%. Third, by realizing high SNR imaging, we succeeded in clearly visualizing local density fluctuations (mottled patterns) of India ink particles, which were buried in the noise floor in conventional low-light imaging.

The ability to detect this “mottled pattern” strongly suggests the possibility of resolving heterogeneous distribution and aggregation phenomena of red blood cells in actual blood flow. The technology established in this study that achieves both wide dynamic range and high SNR is expected to become an extremely important fundamental technology in next-generation non-invasive biomedical imaging devices.

## Figures and Tables

**Table 1:** Main specifications of the prototype chip.

Technology	0.35 μm 2-poly 4-metal standard CMOS
Chip Size	2.07 mm × 2.75 mm
Array Size	128 (H) × 128 (V)
Pixel Pitch	15 μm × 15 μm
Fill Factor	30%
Power Supply	3.3 V, 1.9 V (for comparator)

**Table 2:** Performance comparison between the proposed sensor and state-of-the-art high-dynamic-range sensors for high-precision imaging.

Parameters	This Work	Oikawa [15]	Liu [33]	Miyauchi [34]
Technology	0.35 μm standard	0.18 μm CIS	45 nm/65 nm stacked	45 nm/65 nm stacked
Pixel pitch	15 μm	22.4 μm	4.6 μm	4 μm
Method	Dual-readout self-reset	Three-stage LOFIC	Triple quantization DPS	Two-stage LOFIC
Peak SNR [dB]	>70 dB	69.7 dB	N/A	<50 dB
Max FWC	>14 (=0.78 × 18) Me^−^ *	27.8 Me^−^	9 Me^−^	130 ke^−^
Dynamic range	>92 dB	123 dB	127 dB	90 dB

LOFIC: Lateral Overflow Integration Capacitor. DPS: Digital Pixel Sensor. *: Calculated based on the maximum number of self-resets (17 times) demonstrated in Figure 10.

## Data Availability

The original contributions presented in this study are included in the article/Supplementary Material. Further inquiries can be directed to the corresponding author.

## Supplementary Materials

The following supporting information can be downloaded at: https://www.mdpi.com/article/10.3390/s26041396/s1, Video S1: Difference image video under conditions where self-reset does not occur; Video S2: Difference image video under conditions where the maximum self-reset count reaches 15.

## References

[1] Grinvald A., Hildesheim R. (2004). VSDI: A new era in functional imaging of cortical dynamics. Nat. Rev. Neurosci..

[2] Carlson G.C., Coulter D.A. (2008). In vitro functional imaging in brain slices using fast voltage-sensitive dye imaging combined with whole-cell patch recording. Nat. Protoc..

[3] Ferezou I., Bolea S., Petersen C.C. (2006). Visualizing the cortical representation of whisker touch: Voltage-sensitive dye imaging in freely moving mice. Neuron.

[4] Bouchard M.B., Chen B.R., Burgess S.A., Hillman E. (2009). Ultra-fast multispectral optical imaging of cortical oxygenation, blood flow, and intracellular calcium dynamics. Opt. Express.

[5] Hillman E.M. (2007). Optical brain imaging in vivo: Techniques and applications from animal to man. J. Biomed. Opt..

[6] Tsytsarev V., Liao L.D., Kong K.V., Liu Y.H., Erzurumlu R.S., Olivo M., Thakor N.V. (2014). Recent progress in voltage-sensitive dye imaging for neuroscience. J. Nanosci. Nanotechnol..

[7] Juavinett A.L., Nauhaus I., Garrett M.E., Zhuang J., Callaway E.M. (2017). Automated identification of mouse visual areas with intrinsic signal imaging. Nat. Protoc..

[8] Tsytsarev V. (2023). Optical Imaging of Epileptic Seizures. Handbook of Neuroengineering.

[9] Wu J.Y., Lam Y.W., Falk C.X., Cohen L.B., Fang J., Loew L., Prechtl J.C., Kleinfeld D., Tsau Y. (1998). Voltage-sensitive dyes for monitoring multineuronal activity in the intact central nervous system. Histochem. J..

[10] Zecevic D., Djurisic M., Cohen L.B., Antic S., Wachowiak M., Falk C.X., Zochowski M.R. (2003). Imaging nervous system activity with voltage-sensitive dyes. Curr. Protoc. Neurosci..

[11] http://www.brainvision.co.jp/.

[12] Murata M., Kuroda R., Fujihara Y., Otsuka Y., Shibata H., Shibaguchi T., Kamata Y., Miura N., Kuriyama N., Sugawa S. (2020). A high near-infrared sensitivity over 70-dB SNR CMOS image sensor with lateral overflow integration trench capacitor. IEEE Trans. Electron Devices.

[13] Fujihara Y., Murata M., Nakayama S., Kuroda R., Sugawa S. (2020). An over 120 dB single exposure wide dynamic range CMOS image sensor with two-stage lateral overflow integration capacitor. IEEE Trans. Electron Devices.

[14] Oikawa T., Kuroda R., Takahashi K., Shiba Y., Fujihara Y., Shike H., Murata M., Kuo C.C., Da Silva Y.R.S.C., Goto T. (2022). A 70-dB SNR high-speed global shutter CMOS image sensor for in situ fluid concentration distribution measurements. IEEE Trans. Electron Devices.

[15] Oikawa T., Kuroda R., Hamaya A., Shiba Y., Inada T., Sakai Y., Shirai Y., Sugawa S. (2024). A High SNR Global Shutter CMOS Image Sensor Technology for High Precision Absorption Imaging Applications. ITE Trans. Media Technol. Appl..

[16] Bermak A., Bouzerdoum A., Eshraghian K. (2002). A vision sensor with on-pixel ADC and in-built light adaptation mechanism. Microelectron. J..

[17] Yuan J., Chan H.Y., Fung S.W., Liu B. (2009). An activity-triggered 95.3 dB DR 75.6 dB THD CMOS imaging sensor with digital calibration. IEEE J. Solid-State Circ..

[18] Koppa S., Park D., Joo Y., Jung S. A 105.6 dB DR and 65dB peak SNR self-reset CMOS image sensor using a Schmitt trigger circuit. Proceedings of the 2011 IEEE 54th International Midwest Symposium on Circuits and Systems (MWSCAS).

[19] Park D., Rhee J., Joo Y. (2007). A wide dynamic-range CMOS image sensor using self-reset technique. IEEE Electron. Dev. Lett..

[20] Leñero-Bardallo J.A., Carmona-Galán R., Rodríguez-Vázquez Á. (2017). A wide linear dynamic range image sensor based on asynchronous self-reset and tagging of saturation events. IEEE J. Solid-State Circuits.

[21] Ohta J., Ohta Y., Takehara H., Noda T., Sasagawa K., Tokuda T., Haruta M., Kobayashi T., Akay Y.M., Akay M. (2016). Implantable microimaging device for observing brain activities of rodents. Proc. IEEE.

[22] Ghosh K.K., Burns L.D., Cocker E.D., Nimmerjahn A., Ziv Y., El Gamal A., Schnitzer M.J. (2011). Miniaturized integration of a fluorescence microscope. Nat. Methods.

[23] Ziv Y., Burns L.D., Cocker E.D., Hamel E.O., Ghosh K.K., Kitch L.J., El Gamal A., Schnitzer M.J. (2013). Long-term dynamics of CA1 hippocampal place codes. Nat. Neurosci..

[24] Guo C., Blair G.J., Sehgal M., Sangiuliano Jimka F.N., Bellafard A., Silva A.J., Golshani P., Basso M.A., Blair H.T., Aharoni D. (2023). Miniscope-LFOV: A large-field-of-view, single-cell-resolution, miniature microscope for wired and wire-free imaging of neural dynamics in freely behaving animals. Sci. Adv..

[25] Sasagawa K., Yamaguchi T., Haruta M., Sunaga Y., Takehara H., Takehara H., Noda T., Tokuda T., Ohta J. (2015). An implantable CMOS image sensor with self-reset pixels for functional brain imaging. IEEE Trans. Electron Devices.

[26] Yamaguchi T., Takehara H., Sunaga Y., Haruta M., Motoyama M., Ohta Y., Noda T., Sasagawa K., Tokuda T., Ohta J. (2016). Implantable self-reset CMOS image sensor and its application to hemodynamic response detection in living mouse brain. Jpn. J. Appl. Phys..

[27] Pakpuwadon T., Sasagawa K., Guinto M.C., Ohta Y., Haruta M., Takehara H., Tashiro H., Ohta J. (2021). Self-reset image sensor with a signal-to-noise ratio over 70 dB and its application to brain surface imaging. Front. Neurosci..

[28] Nakamura J. (2005). Image Sensors and Signal Processing for Digital Still Cameras.

[29] Ohta J. (2007). Smart CMOS Image Sensors and Applications.

[30] Bhandari A., Krahmer F., Raskar R. On unlimited sampling. Proceedings of the 2017 International Conference on Sampling Theory and Applications (SampTA).

[31] Bhandari A., Krahmer F., Raskar R. (2020). On unlimited sampling and reconstruction. IEEE Trans. Signal Process..

[32] Mulleti S., Reznitskiy E., Savariego S., Namer M., Glazer N., Eldar Y.C. (2023). A hardware prototype of wideband high-dynamic range analog-to-digital converter. IET Circuits Devices Syst..

[33] Liu C., Bainbridge L., Berkovich A., Chen S., Gao W., Tsai T.H., Mori K., Ikeno R., Uno M., Isozaki T. A 4.6 *μ*m, 512 × 512, ultra-low power stacked digital pixel sensor with triple quantization and 127 dB dynamic range. Proceedings of the 2020 IEEE International Electron Devices Meeting (IEDM).

[34] Miyauchi K., Mori K., Isozaki T., Sawai Y., Chien H.C., Nakamura J. 4.0 μm stacked voltage mode global shutter pixels with a BSI LOFIC and a PDAF capability. Proceedings of the 2021 International Image Sensor Workshop.

